# Resistance exercise leading to failure versus not to failure: effects on cardiovascular control

**DOI:** 10.1186/1471-2261-13-105

**Published:** 2013-11-19

**Authors:** Jéssica Cardoso De Souza, Ramires Alsamir Tibana, Claudia Regina Cavaglieri, Denis César Leite Vieira, Nuno Manuel Frade De Sousa, Felipe Augusto Dos Santos Mendes, Vitor Tajra, Wagner Rodrigues Martins, Darlan Lopes De Farias, Sandor Balsamo, James Wilfred Navalta, Carmen Silvia Grubert Campbell, Jonato Prestes

**Affiliations:** 1 Graduation Program on Physical Education, Catholic University of Brasilia, Brasilia 71966-700, Brazil; 2 Graduation Program Inter-unities - Bioengineering, EESC/FMRP/IQSC, USP, Sao Carlos, Brazil; 3 University of Brasilia, Brasilia, Brazil; 4 Department of Kinesiology and Nutrition Sciences of the University of Nevada, Las Vegas, Nevada, USA; 5 School of Physical Education, State University of Campinas, Campinas, Brazil

**Keywords:** Resistance training, Blood pressure, Hypotension

## Abstract

**Background:**

The aim of the present study was to evaluate the acute effects of resistance exercise (RE) leading to failure and RE that was not to failure on 24 h blood pressure (BP) and heart rate variability (HRV) in sedentary normotensive adult women.

**Methods:**

Ten women (33.2 ± 5.8 years; 159.3 ± 9.4 cm; 58.0 ±6.4 kg; body fat 28.4 ± 2.8%) randomly underwent three experimental sessions: control (40 minutes of seated rest), RE leading to failure with 3 sets of 10 repetitions maximum (10-RM), and RE not to failure at 60% of 10-RM with 3 sets of 10 repetitions. Immediately post session BP and HRV were measured for 24 h.

**Results:**

Ratings of perceived exertion and heart rate were higher during the 10-RM session when compared with 60% of 10-RM (6.4 ± 0.5 vs 3.5 ± 0.8 and 123.7 ± 13.9 vs 104.5 ± 7.3 bpm, respectively). The systolic, diastolic and mean BP decreased at 07:00 a.m. after the 10-RM session when compared with the control session (−9.0 ± 7.8 mmHg, -16.0 ± 12.9 mmHg and −14.3 ± 11.2 mmHg, respectively). The root mean square of the squared differences between R-R intervals decreased after both the 60% of 10-RM and 10-RM sessions compared with the control session.

**Conclusions:**

An acute RE session leading to failure induced a higher drop of BP upon awakening, while both RE sessions reduced cardiac parasympathetic modulation. RE may be an interesting training strategy to acutely decrease BP in adult women.

## Background

Cardiovascular disease is one of the main causes of morbimortality [1], resulting in more than seven million deaths every year [2]. Additionally, there is an association between reduced heart rate variability (HRV), a measure of the cyclic variations of beat-to-beat RR intervals that reflects cardiac autonomic function, and elevated blood pressure (BP) with risk for all-cause mortality [3]. Nevertheless, these numbers can be reduced by changes in lifestyle, such as health diet, the avoidance of smoking and alcohol, as well as regular exercise practice.

It is well established that exercise plays a role as a non-pharmacological tool in the prevention and treatment of several cardiovascular disorders, including both acute and chronic decreases in BP, and modulation of HRV [4-8]. A current challenge for investigators is to delineate the best prescription dose-response for several training modalities in different populations. Additionally, there is a need for studies designed to determine the optimal dose-response for resistance exercise (RE) prescription.

In this sense, RE leading to concentric failure (inability to perform the repetition through the movement’s full range of motion as a consequence of fatigue) has been a target of scientific interest. Among the benefits of performing RE leading to failure are increased motor unit activation [9] and elevated mechanical stress, both associated with an increase of gene expression, muscle damage and consequent muscle repair [10]. However, RE leading to failure may not be the best strategy for optimal strength gains, given that fatigue decreases the capacity of the muscle to generate force [11]. On the other hand, Rooney et al. [12] compared a training protocol utilizing an isoinertial device without rest to another with 30 s of rest between each repetition; the investigators concluded that after six weeks dynamic strength increased by a higher amount in the fatiguing protocol.

Among the possible explanations for these controversial results are the analyzed population (athletes versus physically active individuals), measures of dynamic [12,13] and isometric muscle strength [11], the absence of volume equalization for repetitions, type of device between isokinetic versus isoinercial [11], as well as the lack of training methodologies which reflect RE under practical conditions. Moreover, no investigation has been designed to compare the acute 24 h cardiovascular and autonomic response to a RE protocol leading to failure versus not to failure in untrained individuals. Taking in account that a RE session performed between 8:00-9:00 p.m. induced a reduction of BP during sleep and the first morning hours [8], we have chosen this time frame for RE sessions in the present study.

Considering the higher metabolic and cardiovascular stress of RE leading to failure and the difficulty that untrained individuals might face while performing this type of protocol, the aim of the present study was to evaluate the acute effects of RE leading to failure versus a submaximal protocol (i.e. RE not to failure) on HRV and 24 h BP in sedentary women. The initial hypothesis of the study was RE leading to failure would induce a higher HRV and superior drop on BP during a 24 h period.

## Methods

### Experimental design

A counter-balanced, crossover design was used to compare the effects of RE leading to failure and not to failure on 24 h cardiovascular response in adult women. The importance of RE leading to failure and not to failure remains to be determined, as no specific dose-response has been established regarding this training variable. In the present study, the experimental sessions leading to failure and not to failure, and control session were the independent variables, while the dependent variables consisted of the behavior of 24 h BP and HRV. The difference between experimental RE sessions in this investigation was the training leading to failure and not to failure based on the ten repetitions maximum (10-RM) test. BP and HRV were assessed during a 24 h period to compare the acute effects of RE leading to failure and not to failure. The whole body RE sessions consisted of performing three sets of 10-RM or 60% of 10-RM for each exercise with one minute rest intervals between sets. This design allowed us to individually assess the influence of RE leading to failure in sedentary adult women.

### Subjects

Thirteen women were selected for this study, while one discontinued because of difficulties in traveling to the laboratory, one presented cardiovascular disorders and one was unable to attend the established schedule for the experimental sessions. Thus, ten women (33.2 ± 5.8 yr; 159.3 ± 9.4 cm; 58.0 ±6.4 kg; 28.4 ± 2.8 body fat%; 114.4 ± 11.7 mmHg SBP; 75.1 ± 8.3 mmHg DBP; 79.6 ± 7.8 bpm) were selected for this study. All subjects responded the short-version of the International Physical Activity Questionnaire - IPAQ and were considered sedentary. The inclusion criteria were: pre-menopausal between 24-45 years of age and no regular exercise training in the previous 12 months. The adopted exclusion criteria were: (a) use of drugs that could affect cardiovascular response to RE (i.e., beta-blockers and inhibitors of angiotensin-converting enzyme), (b) smoking and (c) presence of any cardiovascular or osteomyoarticular problems that could affect performance during RE.

Volunteers completed a thorough physical examination, including a medical history, resting electrocardiogram, BP assessment, anthropometric, and orthopaedic evaluation prior to participation in the experimental sessions. Moreover, all volunteers signed a written consent and were informed about the risks and benefits of the present study which was approved by the Catholic University of Brasilia Research Ethics Committee for Human Use (Protocol number 376/2010).

### Anthropometrics, body fat percentage and hemodynamic parameters

Height was measured by a wall-stadiometer (Sanny, Sao Paulo, Brazil), with a capacity of 2200 mm and precision of 1 mm. Weight was determined on a digital scale (Welmy-W110H, Sao Paulo, Brazil). Body fat percentage was determined by the Jackson and Pollock seven-site skinfold protocol [14]. Skinfold thickness was measured at seven sites: sub-scapular, triceps, biceps, chest, abdomen, thigh and suprailiac, by a Lange skinfold caliper (Beta Technology Inc, Santa Cruz, CA, USA). Three measurements were made of each skinfold, and the average value was used to calculate body fat percentage. Systolic (SBP) and diastolic blood pressure (DBP) were measured with a validated oscillometric device (Microlife 3 AC1-1, Widnau, Switzerland). Blood pressure was assessed in triplicate (measurements separated by 5 min) with the mean value used for further analysis after 10 minutes of seated rest. Heart rate (HR) was measured by a HR monitor (Polar® S810i, Polar Electo Oy, Kempele, Finland). During BP and HR monitoring, participants remained in a seated position in a temperature controlled, quiet room (23°C).

### 10-RM testing

Individuals completed two weeks of familiarization prior to testing. During the familiarization weeks individuals were advised regarding proper technique execution and completed 3 sessions/week, with one exercise for each main muscle group with 3 sets of 10-12 submaximal repetitions. After the familiarization period a 10 repetition maximum (10-RM) test and re-test was performed to determine the exact training load for each exercise on two nonconsecutive days with 48-72 hours between tests. The 10-RM test exercises were randomly performed to avoid an effect of exercise order for the following exercises: chest press, leg press, front lat pull-down, leg extension, triceps pulley, leg curl, biceps curl and seated calf raise (Jonhson, Landmark Drive, Cottage Grove, USA). Prior to testing, subjects performed 5 min of low intensity walking on a treadmill. The 10-RM testing procedures progressed as follows: 1) warm-up on each RE with 5-10 submaximal repetitions using a light load (60% of the predicted 1-RM); 2) 1 minute rest and load increments of 5-10% until the 10-RM was found within 3-5 attempts, using 3-5 minute rest intervals between them; 3) subjects were instructed to lift and lower the load at a constant velocity, taking approximately 2 s for each phase of the movement; 4) ten repetitions were recorded with the maximal load determined by the last successful repetition with individual supervision [15]. Test/re-test reliability for the 10-RM was performed and a high intraclass correlation coefficient (ICC) was found, R = 0.98 for all tested exercises.

The following strategies were adopted to minimize testing errors: a) all subjects participated in a familiarization period prior to testing, b) standardized instructions were provided to all subjects before the test, c) subjects were carefully instructed about maintaining proper exercise technique and body position d) consistent verbal encouragement was provided during the testing procedures to all subjects. All testing sessions were scheduled between 8:00-9:00 p.m.

### Twenty four hour blood pressure measurement

All testing and training sessions were conducted between 08:00-09:00 p.m. In the control session without exercise and immediately after the RE sessions subjects underwent 24 h BP monitoring (Dyna-MAPA, Cardios, Sao Paulo, Brazil). All subjects received instructions about the technique and position of the cuff during the 24 h BP monitoring. In addition, the subjects were encouraged to avoid smoking, alcohol, caffeine, unusual physical activity and to maintain their usual diet consumption (this was confirmed by a dietary recall follow-up). Individuals were asked to go to bed at 11:00 p.m. and awaken at 06:00 a.m. on recording days. They were also advised to have a meal two hours before testing to avoid a fasted state.

As previously published [8] 24 h BP was measured on the non-dominant arm with an oscillometric monitor (Dyna-MAPA, Cardios, Sao Paulo, Brazil) in accordance with manufacturer’s instructions. The monitor was programmed to perform measures every 15 minutes during the daytime and every 30 minutes during sleeping hours. All measures of BP were performed during weekdays (i.e. Tuesday or Thursday), and initiated between 9:00-10:00 p.m. All participants were advised to maintain their habitual activities, refrain from programmed exercise at least 72 hours before the measures, and to stop and relax the arm during each measurement. Data were calculated and analyzed as follows: mean of all measurements during the 24 h period (24 h), and mean of all measures performed during daytime and sleeping hours. The BP measurements were considered invalid for analysis if >30% of the measurements were missing, if data were lacking for an interval of >2 h, or if the sleep period was <6 or >12 h. Liquid ingestion was controlled before data acquisition and during the 24 h period of BP measure. Additionally, the cuff size was adapted to the circumference of the arm of each participant according to the manufacturer’s recommendations.

### Heart rate variability measurement

The autonomic modulation of HR was obtained from the spectral analysis of R-R intervals obtained from a heart rate monitor (Polar, S810i, Kempele, Finland) during a 24 h period immediately after the end of the RE sessions and control session. R-R intervals were recorded at a sampling rate of 1 kHz with all intervals manually inspected to exclude artifacts with corresponding software prior to analysis. Stationary periods with at least 500 beats were analyzed with default values using custom designed software (Kubios v2., University of Kuopio, Finland) in accordance with the recommendations of the Task Force (1996) to identify the sub-bands of low frequency (LF) (0.04-0.15 Hz) and high frequency (HF) (0.15-0.4Hz). HF and LF normalized reflect parasympathetic modulation. LF/HF ratio signifies the overall balance between sympathetic and parasympathetic systems. The power of each spectral component was normalized (n.u.) for analysis as previously described [16]. The root mean square of the squared successive differences between the adjacent R-R intervals (rMSSD), which reflects parasympathetic regulation of the heart, was used for analysis. The HRV can be measured by two methods: the time domain method and the frequency domain method. For the time domain method we used rMSSD and for the frequency domain method we used LF, HF and LF/HF ratio [16].

### Resistance exercise sessions

After the two weeks of familiarization to minimize excessive muscle soreness, five sessions were performed on nonconsecutive days with a 48-72-hour rest interval between sessions. On days one and two, subjects performed a 10-RM test and re-test, respectively. Days three, four and five were randomly dedicated to RE sessions leading to failure or not to failure and control session. Whole body RE sessions consisted of the following exercises: chest press, leg press, front lat pull-down, leg extension, triceps pulley, leg curl, biceps curl and seated calf raise. In the failure protocol, subjects completed three sets of each exercise to volitional exhaustion using the predetermined 10-RM load in each exercise. The RE session leading to failure followed the recommendations of the American College of Sports Medicine [17] for muscle hypertrophy in untrained individuals. If necessary, individuals were spotted to complete 10-RM in each set. In the protocol that was not to failure, subjects performed three sets of ten repetitions in each exercise using using 60% of 10-RM. The rest interval between sets and exercises was one minute. Subjects were instructed to perform each repetition at a moderate velocity (i.e., 2 sec concentric and 2 sec eccentric). All training sessions were scheduled between 8:00-9:00 p.m. Heart rate was assessed with a heart rate monitor (Polar FT 1, Kempele, Finland) after each set of the first exercise, the middle session, and upon completion of the entire RE bout. Rating of perceived exertion (RPE) was assessed after the last RE set following instructions described by Robertson et al. [18]. The OMINI scale has specific descriptors distributed along a comparatively narrow numerical response range (0-10), and is presented in a visually discernible exertional intensity gradient [18]. The scale also provides verbal descriptors along with the narrow numerical range and was designed to subjectively evaluate the individual perceived exertion to a RE protocol [18]. Additionally, subjects were advised regarding correct breathing patterns and to avoid the Valsalva maneuver (VM), because BP is significantly higher when performing a VM compared with free breathing. For the control session, subjects visited the laboratory between 8:00-9:00 p.m. and the measures of BP and HRV collected at the same time as the RE sessions without performing any type of exercise.

### Statistical analysis

The data are expressed as means ± SD. The Shapiro-Wilk test was applied to check for normality of the variables. In the case of a non-normal distribution, logarithmic transformation was performed. Paired t tests were applied to compare RPE and heart rate during the RE sessions. One-way ANOVA was used to compare the BP and HRV between the control and RE sessions. Tukey’s post-hoc test was applied in the event of significance. Considering a power (1-β) of 0.80 and an alpha error of 0.05, the sample size used in this research allowed identifying a large effect size (*f*^2^ = 0.60). The level of significance was *p* ≤ 0.05 and SPSS version 20.0 (Somers, NY, USA) software was used.

## Results

Systolic and diastolic blood pressure at rest was considered to be within normal range. The training sessions were well controlled so that all subjects performed within the prescribed workload for each RE session (Table 1). The intensity zones defined by 10-RM seemed to accurately demarcate two different training zones based on RPE and HR. Both RE sessions induced a substantial increase in HR, with a higher increase during the 10-RM RE session when compared with 60% of 10-RM (*p* ≤ 0.05). Similarly, higher RPE values were found for 10-RM (*p* ≤ 0.05; Table 2).

**Table 1:** Workload (mean ± SD) for each resistance exercise

	**Chest press**	**Leg press**	**Frontal lat pull-down**	**Leg extension**	**Triceps pulley**	**Leg curl**	**Biceps curl**	**Seated calf rise**
60% of 10-RM, kg	20.9 ± 4.6	40.9 ± 10.8	17.4 ± 2.2	23. ± 6.4	16.7 ± 3.5	17. ± 5.3	15.1 ± 2.5	8.1 ± 1.8
10-RM, kg	34.8 ± 7.7	68.2 ± 18.0	28.9 ± 3.7	39.2 ± 10.5	27.8 ± 5.9	28.6 ± 8.8	25.2 ± 4.2	13.4 ± 3.0

10-RM = ten repetitions maximum.

**Table 2:** Heart rate and ratings of perceived exertion (RPE) during the experimental sessions (mean ± SD)

	**RPE**	**Heart rate, bpm**		
		**1st exercise**	**Middle session**	**Last exercise**
60% of 10-RM	3.5 ± 0.8	108.5 ± 14.2	104.9 ± 13.0	104.5 ± 7.3
10-RM	6.4 ± 0.5*	134.4 ± 18.4*	129.7 ± 15.6*	123.7 ± 13.9*

*p ≤ 0.05 vs 60% of 10-RM session. 10-RM = ten repetitions maximum.

**Figure 1:**
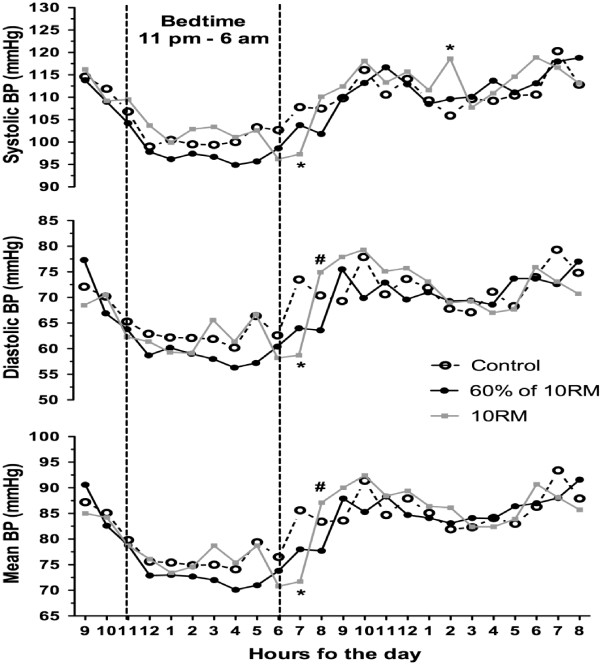
**Systolic, diastolic and mean blood pressure (BP) measures during 24 h after 60% of 10-RM and 10-RM resistance exercise sessions and control session.** *p ≤ 0.05 vs control session; #*p* ≤ 0.05 vs 60% of 10-RM session. 10-RM = ten repetitions maximum. Values are presented as means.

**Figure 2:**
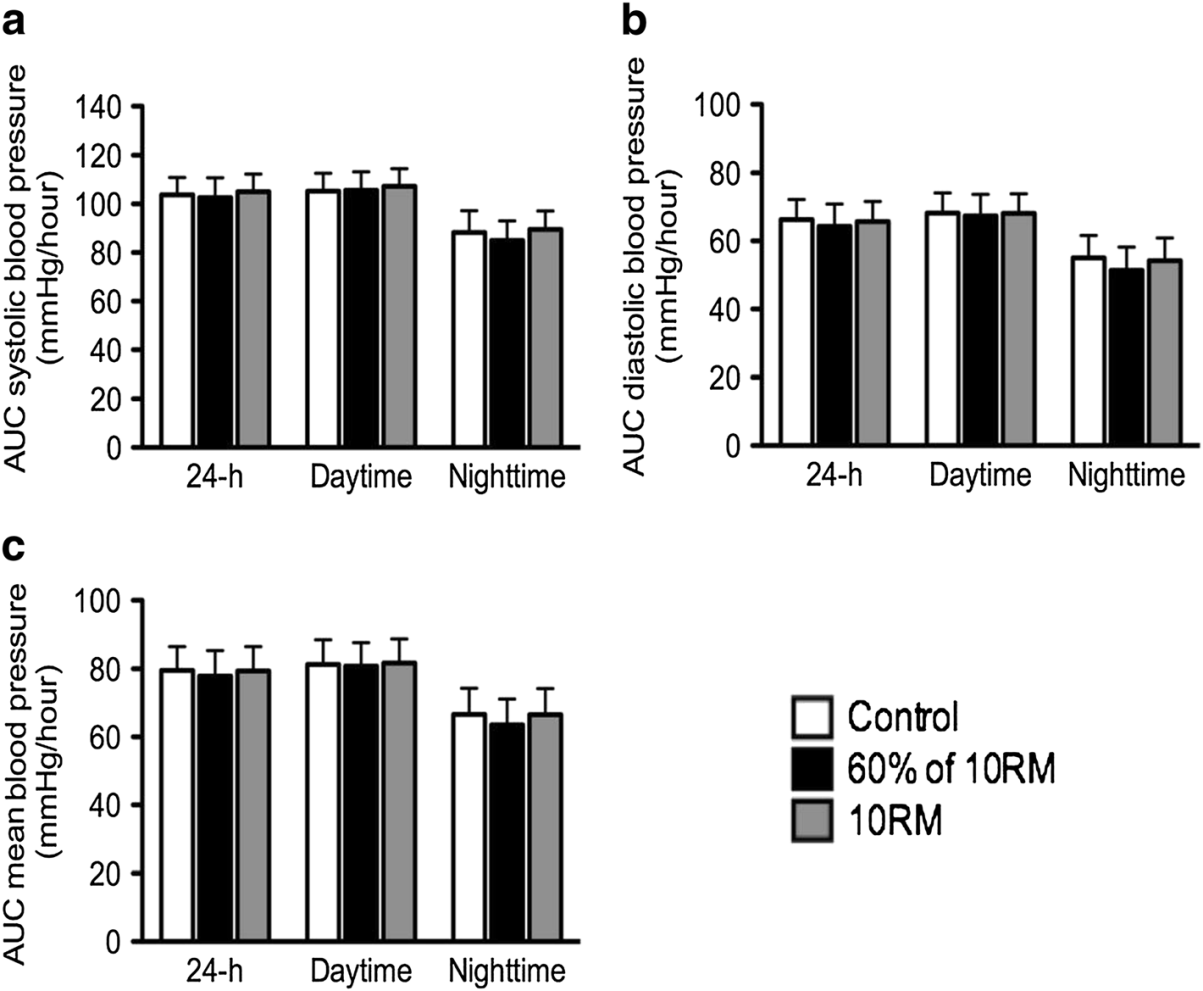
**Twenty four hour, daytime and nighttime areas under the curve (AUC) of blood pressure after 60% of 10-RM and 10-RM resistance exercise sessions and nonexercise control period (control).** Systolic blood pressure **(a)**; diastolic blood pressure **(b)** and mean arterial pressure **(c)**. 10-RM = ten repetitions maximum. Values are presented as mean ± SD.

Figure 1 presents the mean values of SBP, DBP and MBP during each hour of sleep and awake conditions. The SBP, DBP and MBP presented a significant decrease (*p* ≤ 0.05) at 07:00 a.m. after the 10-RM session when compared with the control session (−9.0 ± 7.8 mmHg, -16.0 ± 12.9 mmHg and −14.3 ± 11.2 mmHg, respectively). On the other hand, DBP and MBP were higher (*p* ≤ 0.05) at 08:00 p.m. following the 10-RM session when compared with 60% of 10-RM (11.1 ± 9.2 mmHg and 8.9 ± 7.9 mmHg, respectively). The SBP increased (*p* ≤ 0.05) after the 10-RM session at 02:00 p.m. (12.7 ± 7.6 mmHg) as compared with the control session. There were no differences for the areas under the BP curve of 24 h, daytime and nighttime in the non-exercise control session, after the 10-RM and 60% of 10-RM sessions (*p* ≥ 0.05; Figure 2).

The time and frequency domain of HRV during the periods of 8:00 p.m. to 10:00 a.m., 8:00 p.m. to 6:00 a.m. and 7:00 a.m. to 10:00 a.m. are presented in Table 3. The rMSSD decreased (*p* ≤ 0.05) after 60% of 10-RM and 10-RM sessions compared with the control session during the three evaluated periods. There was no significant difference in rMSSD between RE sessions. Frequency domain measures of HRV remained unchanged (*p* ≥ 0.05) during all experimental conditions. Although not statistically different, there was a tendency toward an increase in LF and LF/HF ratio and a decrease in HF parameters after the RE sessions.

**Table 3:** Heart rate variability (mean ± SD) parameters in the control and experimental sessions

	**8 pm – 10 am**	**8 pm – 6 am**	**7 am – 10 am**
Control session			
rMSSD (ms)	68.5 ± 31.2	67.4 ± 34.3	72.2 ± 28.9
LF n.u. (%)	68.8 ± 8.7	70.0 ± 8.8	62.0 ± 11.9
HF n.u. (%)	31.2 ± 8.7	30.0 ± 8.8	37.9 ± 11.9
LF/HF	3.0 ± 1.8	3.2 ± 1.9	2.0 ± 1.0
60% of 10-RM session			
rMSSD (ms)	39.0 ± 16.8*	38.8 ± 17.3*	33.9 ± 15.4*
LF n.u. (%)	68.4 ± 12.3	67.8 ± 12.0	74.3 ± 14.6
HF n.u. (%)	31.6 ± 12.3	32.2 ± 12.0	25.7 ± 14.6
LF/HF	3.1 ± 1.3	2.9 ± 1.1	4.2 ± 2.8
10-RM session			
rMSSD (ms)	43.6 ± 17.7*	41.8 ± 18.0*	44.7 ± 17.5*
LF n.u. (%)	69.6 ± 4.6	68.1 ± 5.8	74.1 ± 7.6
HF n.u. (%)	30.4 ± 4.6	31.9 ± 5.8	25.9 ± 7.6
LF/HF	3.0 ± 0.6	2.8 ± 0.8	3.7 ± 1.5

*p ≤ 0.05 vs control session. rMSSD = root mean square of the squared successive differences between the adjacent R-R intervals; LF = low-frequency component; HF = high-frequency component (rMSSD, LF and HF reflecting a parasympathetic regulation). LF/HF ratio signifies the overall balance between sympathetic and parasympathetic systems.10-RM = ten repetitions maximum.

## Discussion

The aim of the present study was to evaluate the acute effects of RE leading to failure and not to failure on 24 h BP and HRV in sedentary women. The results indicate that a RE session leading to failure induced a drop in BP upon awakening (at 07:00 a.m.) when compared with the control session. Additionally, the RE leading to failure and not to failure decreased the rMSSD (parasympathetic regulation) during the periods of 8:00 p.m. to 10:00 a.m., 8:00 p.m. to 6:00 a.m. and 7:00 a.m. to 10:00 a.m. compared with the control session. Furthermore, RE leading to failure induced a substantial increase in HR and RPE during and immediately after the RE when compared with 60% of 10 RM.

It is well established that exercise plays a role as a non-pharmacological tool in the prevention and treatment of several cardiovascular disorders, including both acute and chronic decreases in blood pressure [4-8,19,20]. For example, Tibana et al. [20] found that an acute submaximal RE session was effective in decreasing SBP, DBP and MBP during 24 h and throughout nighttime hours in overweight/obese middle-aged women [8]. In a chronic study, Strasser et al. [19] found a significant reduction of mean BP measured during 24 h after a 4-month RE program in patients with type 2 diabetes.

To best of our knowledge, this is the first study to evaluate the effect of RE leading to failure and not to failure on HRV and 24 h BP in sedentary normotensive women. Studies have shown that after the performance of an acute RE bout, SBP or DBP may be elevated [21], reduced [7,8,22,23] or unchanged [24,25] in comparison to pre-exercise or control measurements. In the present study, BP reduction (upon-awakening) was observed just after the RE session leading to failure when compared with the control session. In this sense, Polito et al. [22] also reported a significant post-exercise reduction in SBP after two RE sessions with different intensities. Similar to that observed in the present study, no significant differences between the protocols were found regarding the magnitude of post-exercise hypotension (PEH). However, PEH lasted longer after the most intense protocol (6RM).

In a similar study, Simão et al. [23] compared the effect of intensity, volume and session format on PEH response. Significant post-exercise reduction of SBP lasting up to 50-60 minutes was found after the protocols. No significant differences between the protocols were observed regarding the magnitude of PEH. Thus, one can hypothesize that the results of the present study can be, at least in part, explained by the difference in the intensity between RE sessions. In fact, the RE session leading to concentric failure induced a higher increase of RPE and HR when compared with the RE session not to failure. MacDonald [26] proposed that the release of substances (eg, potassium, adenosine, nitric oxide, etc) after exercise is one of the main factors involved in muscle vasodilatation and the fall of peripheral vascular resistance.

On the other hand, the present results are different from the results of Scher et al. [27] that verified a reduction of BP during the first 60 minutes and during 24 h after RE with low-intensity (40% of 1-RM), and Queiroz et al. [28] that verified a reduction in SBP, DBP and MBP under clinical, but not ambulatory, conditions after low-intensity RE (50% of 1-RM). Moreover, previous studies with high-intensity RE observed no decrease of SBP or DBP during the recovery period [24,29,30], and some even have reported an increase in SBP [21].

The application of the RE session between 8:00-9:00 p.m., as in the present study was favorable to induce the reduction of BP in the first morning hours. This is of great relevance, considering that the MAPEC study reported that taking ≥ 1 hypertensive medication before sleeping promotes greater cardiovascular protection in comparison to the use of these medications upon awakening [31]. In addition, the occurrence of acute myocardial infarction, sudden cardiac death, pulmonary embolism, critical limb ischemia, and aortic aneurysm rupture, are more susceptible to happen upon peak awakening in the morning and during the secondary early evening peak [31].

The main difficulties in comparing our results with previously published studies are that most studies were limited regarding the time-course analyses of BP and different exercise protocols were used. To note, the RE completed in the present study is widely used in daily practice, as we selected one RE for each main muscle group, which reinforces the clinical relevance of the results. Additionally, the application of 24 h BP monitoring has strong correlation with overall target organ damage score, left ventricular mass, impaired left ventricular function, albuminuria, brain damage and microvascular disease, and especially retinopathy [32].

The underlying mechanisms responsible for the training-induced reduction in BP remain unclear. Despite the fact that the decline in BP is likely multifactorial, some proposals indicate a decreased cardiac output and peripheral vascular resistance due to lowered sympathetic activity, inducing transduction for vascular tone [33-35] higher activity of the plasma kallikrein system mediating nitric oxide release [36], alterations in cerebral blood flow induced by exercise [34] and findings from recent studies include the recognition that sustained post-exercise vasodilation of the previously active skeletal muscle is primarily the result of histamine H1- and H2-receptor activation [37].

In the current study, RE leading to failure and not to failure concentric failure caused a reduced cardiac parasympathetic modulation compared with the control session during the three evaluated periods (8:00 p.m. to 10:00 a.m., 8:00 p.m. to 6:00 a.m. and 7:00 a.m. to 10:00 a.m.). These results are similar to those observed by Rezk et al. [29] who observed similar changes in cardiac parasympathetic modulation after the completion of RE with 80% of 1-RM and 40% of 1-RM leading to failure. On the other hand, different from the present study, the results observed by Lima et al. [4] revealed that RE for the trunk and upper limbs induced an increased cardiac sympathetic modulation and reduced cardiac parasympathetic modulation. Moreover, this change in the cardiac autonomic modulation was higher after a more intense RE (70% of 1-RM vs 50% of 1-RM) [4]. Similarly, Simões et al. [38] observed a significant decrease in the standard deviation of the instantaneous beat-to-beat R-R interval variability (SD1) and rMSSD indexes beginning at 30% 1-RM during leg RE that was related to lactate. They concluded that this intensity (30% of 1-RM) was associated with the point at which aerobic metabolism becomes supplemented by anaerobic metabolism. However, in the present study the RE session performed with 10-RM induced a similar response regarding the sympathetic and parasympathetic modulation as compared with the RE session not to failure [38]. Moreover, different from the studies by Rezk et al., [29], Simões et al., [38], Lima et al., [4] and Tibana et al., [7] who analyzed the HRV for few minutes after exercise, in the present study we analyzed HRV after RE for several hours.

This study has limitations that should be considered. The small sample size that was utilized and the individuals in this study were adult, limiting the extrapolation of these results to individuals with other characteristics. Moreover, post exercise hypotension mechanisms were not investigated. Additionally, blood sample measures should be considered for future studies, including the analysis of metalloproteinases. The assessment of energy expenditure during BP measuring days could provide more precise information regarding their activities following RE sessions.

## Conclusions

In summary, the findings of the present study revealed that, in normotensive adult women an acute RE session leading to failure induced a drop of BP upon-awakening (at 07:00 a.m.). RE may be an interesting training strategy to acutely decrease BP in adult women. Moreover, RE leading to failure as well as not to failure decreased cardiac parasympathetic modulation. We suggest further studies to evaluate the effects of RE leading to failure and not to failure on HRV and 24 h BP in different populations such as the elderly, hypertensive subjects, and diabetic individuals. Another future direction would be to investigate the chronic effects of resistance training leading failure on BP. Additionally, the physiological mechanisms involved in the superior post-resistance exercise hypotension require further investigation.

## Competing interests

The authors declare that they have no competing interests or non-financial competing interests that may cause embarrassment to become public after the publication of the manuscript.

## Authors’ contributions

JCS, RAT and JP: study idealization and design, data collection, writing of the introduction, results, discussion and conclusion. DCLV, FASM, WRM, DLF and VT: participation in the selection of the individuals, study idealization, data collection and methods. NMFS: participated of the statistical analysis and writing of the methods. SB, JWN, CSGC and CRC: participated in the study design, data analysis, and writing of the results and discussion. All authors read and approved the final manuscript.

## Pre-publication history

The pre-publication history for this paper can be accessed here:

http://www.biomedcentral.com/1471-2261/13/105/prepub
